# The proteoglycan repertoire of lymphoid cells

**DOI:** 10.1007/s10719-012-9427-9

**Published:** 2012-07-10

**Authors:** Bodil Fadnes, Anne Husebekk, Gunbjørg Svineng, Øystein Rekdal, Masaki Yanagishita, Svein O. Kolset, Lars Uhlin-Hansen

**Affiliations:** 1 Institute of Medical Biology, Faculty of Health Sciences, University of Tromsø, Tromsø, Norway; 2 Division of Diagnostic Services, University Hospital of North Norway, Tromsø, Norway; 3 Lytix Biopharma, Tromsø Science Park, Tromsø, Norway; 4 Tokyo Medical and Dental University, Tokyo, Japan; 5 Department of Nutrition, University of Oslo, Oslo, Norway

**Keywords:** Proteoglycan, Serglycin, Syndecan, Glypican, Lymphoid cells

## Abstract

Proteoglycans have been studied to a limited extent in lymphoid cells. In this study we have investigated the expression of proteoglycans in B-cells, CD4+ T-cells, CD8+ T-cells, natural killer cells, as well as in nine different cell lines established from patients with lymphoid malignancies. Serglycin was the major proteoglycan expressed at mRNA level by the primary lymphocytes. None of the syndecans or glycpicans was detected at mRNA level in the primary lymphocytes, except for syndecan-4 in CD4+ T-cells and CD8+ T-cells. All lymphoid cell lines expressed serglycin mRNA, as well as one or several members of the syndecan and glypican families. Further, increased synthesis of proteoglycans was found in the cell lines compared to the primary lymphocytes, as well as the presence of heparan sulfate on the cell surface of five of the cells lines. Western blot analysis showed a close correlation between serglycin mRNA level and expression of serglycin core protein. Our results show that serglycin is a major proteoglycan in all the normal lymphoid cells and that these cells carry little, or none, proteoglycans on the cell surface. Serglycin was also a major proteoglycan in the malignant lymphoid cells, but these also expressed one or more types of cell surface proteoglycans. Thus, malignant transformation of lymphoid cells may be followed by increased synthesis of proteoglycans and expression of cell surface proteoglycans.

## Introduction

Proteoglycans (PGs) constitute a diverse group of glycoconjugates, characterized by one or more highly sulfated glycosaminoglycan (GAG) chain(s) covalently linked to a core protein [1]. PGs are found in all types of tissues and can be localized in extracellular matrix, on the cell surface [2], and in intracellular granules [3].The biological functions of the PGs are usually closely linked to the structure of the GAG side chains, of which heparan sulfate (HS) and chondroitin sulfate (CS) are the two major types [1,4]. Both types of GAGs are known to interact with a large variety of molecules, but in general a much higher affinity is evident for HS compared to CS. The superior binding capacity of HS compared to that of CS has been ascribed to the unique sulfation pattern and a higher degree of conformational flexibility of the HS chains [5]. Serglycin is a PG which is found in intracellular granules of various cell types, including macrophages, mast cells, neutrophils, platelets, cytotoxic T-lymphocytes and endothelial cells [3,6]. Human serglycin can potentially carry eight GAG chains, which in most cases are CS chains. However, serglycin from connective tissue mast cells mostly have heparin chains attached to its core protein [3,7]. Serglycin has been shown to play a key role in promoting the storage, and in regulating the activities, of a number of proteases [6]. Most cell types have one or more species of PG at the cell surface. The most abundant cell surface PGs belong to the syndecan and glypican families. So far, 4 different syndecans and 6 different glypicans have been characterized [8]. Syndecans are integral membrane proteins, whereas glypicans are linked to the cell surface by a glycosylphosphatidylinositol anchor. The syndecans contain 3–5 HS chains attached to conserved motifs in the ectodomain, although hybrid forms with both HS and CS are also found [9]. The glypicans contain 2–3 attachment sites for HS chains [10]. Cell surface HS binds a variety of extracellular-matrix molecules and growth factors and are therefore crucial for normal physiology [11]. Several reports indicate that cell surface HS PGs also are involved in growth and metastasis of malignant cells and abnormal expression patterns of both syndecans and glypicans have been demonstrated in several cancers [12].

The knowledge of PG expression in various types of lymphoid cells remains fragmented. Syndecan-1 is expressed at the cell surface of plasma cells [13], but has not been reported on other normal lymphoid cells. Syndecan-2 and -4 have been found on activated T-cells [14,15], whereas serglycin has been reported to be the major PG in CD8+ T lymphocytes [16].

Not much is known about the expression of PGs in lymphomas and leukemias. Syndecan-4 has recently been reported to be expressed by malignant T-cells in patients with Sézary syndrome [17]. It was speculated that syndecan-4 plays a pathogenic role in this syndrome and is a possible target for treatment. Detection of syndecan-1 has become a useful tool in the diagnosis of some hematological malignancies, since syndecan-1 is expressed in plasmacytoma/plasma cell myeloma, but absent in most types of non-Hodgkin lymphomas [18]. Further, Reed Sternberg cells in classical Hodgkin disease express syndecan-1, whereas the putative tumor cells of nodular lymphocyte predominant subtype of Hodgkin disease do not [19]. Serglycin has been demonstrated to be a marker of acute myeloid leukemia (AML) [20].

In the present study we wanted to investigate the expression of syndecans, glypicans and serglycin in various types of normal lymphoid cells, as well as in different lymphoma/leukemia cell lines. For this purpose we have used cell sorting, RT-qPCR, flow cytometry, radiolabelling and GAG structure analyses. Our results show that serglycin was the dominant PG expressed by normal B- and T-lymphocytes. Serglycin was also a major PG in the malignant lymphoid cells, but these cells also expressed one or more types of cell surface PGs.

## Materials and methods

### Materials

[^35^ S]Sulfuric acid was purchased from Du Pont-New England Nuclear. Superose 6 (HR 10/30), Q-Sepharose and Sephadex G-50 (fine) were obtained from Amersham Pharmacia Biotech (Uppsala, Sweden). Chondroitin sulfate ABC lyase (cABC) (*Proteus vulgaris*), heparitinase (*Flavobacterium heparinum*) and FITC conjugated anti-HS antibody (10E4) were obtained from Seikagaku Kogyo Co (Tokyo, Japan). Phytohemagglutinin-L (PHA-L) was purchased from Roche Applied Science (Oslo, Norway). Antibody-coated magnetic beads were from Invitrogen (DynalBiotech, Oslo, Norway). FITC-, PE- and PerCP-conjugated anti- CD45, -3, - 4, -8, -19, -56 and anti-CD138 (syndecan-1) antibodies were obtained from Becton Dickinson (San Jose, CA, USA). MagicMarker and NuPage Novex 4–12 % Bis-Tris gels were from Invitrogen (Carlsbad, CA, USA). The antibody against serglycin was prepared as described previously [21]. HPR-linked anti-rabbit antibody (#4050-05) was obtained from SouthernBiotech (Birmingham, AL, USA), while the Western Blotting Luminol Reagent was obtained from Santa Cruz Biotechnology Inc., (Santa Cruze, CA, USA).

### Cell lines

The myeloma cell line U-266 was purchased from ATCC. The Burkitt lymphoma cell lines Namalwa and Ramos, and the T cell lymphoma cell lines Sup-T, CEM, H9 and MT-4 were all obtained from Dr. Michael Norcross, Center of Drug Evaluation and Research, Food and Drug Administration, Bethesda, MD, USA. The B cell line SUDHL-6 and the myeloma cell line KMS-5 were kindly provided by Dr. Mark Raffeld, National Cancer Institute, National Institute of Health, Bethesda, MD, USA. All cell lines were cultured in RPMI 1640 with 10 % fetal bovine serum.

### Isolation and culture of primary cells

Lymphocyte-enriched cell suspensions were isolated from peripheral blood by density gradient centrifugation and counterflow centrifugal elutriation using a Model J-6 M centrifuge (Beckman Instruments, Palo Alto, CA, USA) equipped with a JE-10X elutriation rotor, as previously described [22]. The purity of the lymphocytes was greater than 98 % as determined by flow cytometry. CD4+ T-cells were prepared by negative selection of CD8+ T-cells and CD19+ B-cells, CD8+ T-cells by negative selection of CD4+ T-cells and CD19+ B-cells, B-cells by negative selection of CD4+ and CD8+ T-cells, and NK-cells by negative selection of CD4+ T-cells, CD8+ T-cells and CD19+ B-cells. The different cells obtained were cultured in RPMI 1640 containing 10 % fetal bovine serum. In some experiments CD4+ and CD8+ cells were stimulated with 1 μg/ml PHA-L for 20 h.

### Metabolic labeling and isolation of radiolabeled macromolecules

After negative selection separate cultures of CD4+ T-cells, CD8^+^ T-cells, NK-cells and B-cells, as well as all the B- and T-cell lines, were incubated in the presence of [^35^ S]sulfate (100 μCi/ml) for 20 h in RPMI 1640 with 10 % fetal bovine serum. After radiolabeling, the culture medium was separated from the cells by centrifugation. The cells were then solubilized with 4 M guanidine HCl containing 2 % Triton X-100. Guanidine HCl was also added to the culture medium fractions to a final concentration of 4 M. Unincorporated [^35^ S]sulfate was removed from the samples by chromatography on Sephadex G-50 (fine) columns [23].

### Enzymatic and chemical treatment

Digestions of radio labeled macromolecules with cABC (0.01 unit/ml) or heparitinase (0.01 unit/ml) were done in 0.1 M Tris HCl, 0.1 M sodium acetate, pH 7.3, at 37 °C for 2 h. Nitrous acid treatment at pH 1.5, was performed as described by Shively and Conrad [24]. Alkaline borohydride treatment was done by incubating the sample in 0.1 M NaOH, containing 1 M NaBH_4_, at 45 ˚C. After 24 h the samples were neutralized by adding 5 M acetic acid.

### Gel chromatography and quantification of HS and CS

Radiolabeled samples subjected to different types of treatment were analyzed by gel chromatography on a Superose 6 gel chromatography column run in 4 M guanidine HCl, 0.1 M sodium acetate, 0.5 % Triton X-100, pH 6.0, at a flow rate of 0.4 ml/min. Fractions of 1 min were collected and analyzed for radioactivity in a scintillation counter after the addition of Ultima Gold XR scintillation fluid. The amounts of CS and HS in the respective fractions were calculated after gel chromatography. Material eluting in positions close to the V_t_ elution position after cABC treatment was taken as a measure of CS amount. The same was done for material eluting in retarded positions after heparitinase or nitrous acid treatment to determine HS amounts in ^35^ S-labeled macromolecules recovered from medium and cells fractions from labeled primary cells or cell lines.

### Western blot analysis

Serum free medium from the cell lines KMS-5, U-266, Namalwa and Ramos, cultured for 20 h, was treated with cABC for 2 h at 37 °C before the samples were run on NuPage Novex 4–12 % Bis-Tris gels. A protein ladder (MagicMark) was used as a molecular weight maker. Serglycin purified from human myeloma cell lines, kindly provided by Dr. Theocharis (Laboratory of Biochemistry, Department of Chemistry, University of Patras, Greece) [21] was treated with cABC for 2 h at 37 °C and used as a positive control. Samples were blotted onto a polyvinyl difluoride (PVDF) membrane, blocked in 5 % non-fat dry milk in TBS with 0,1 % Tween 20 and probed with anti-serglycin, followed by HPR-linked anti-rabbit antibody. Western Blotting Luminol Reagent was used for detection. The intensity of immunoblot bands were measured using a Luminescent Image ANalyzer LAS-300 with MultiGauge software version 3.0 (Fujifilm, Tokyo, Japan).

### Flow cytometry

Anti-CD138/syndecan -1 and anti-HS (10E4) antibodies were used to investigate the possible presence of cell surface PGs (syndecan-1 and HS). Peripheral Blood Mononuclear Cell (PBMC) were isolated from fresh peripheral blood. All cells were stained with fluorescent conjugated antibodies and were sorted and counted in a flow cytometer (FACSAria, Becton Dickinson, San Jose, CA). The following fluorescence conjugated antibodies were used to identify the different cells; B-cells (CD19-AC-Alexa-750 pos; CD3-FITC and CD14-PerCP neg), T-cells (CD3-APC, CD4-FITC or CD8-PE-Alexa-700 pos; CD14APC-Alexa-750 and CD19 APC-Alexa-750 neg) and NK cells (CD56-FITC pos; CD19-APC-Alexa-750, CD14-APC-Alexa.750 and CD3-APC neg).

### RNA extraction

Total RNA was extracted from freshly isolated subsets of human lymphocytes (CD4+ T-cells, CD8+ T-cells, NK-cells and B-cells) isolated by cell sorting and from cultured cell lines (Namalwa, Ramos, SUDHL-6, U-266, KMS-5, Sup-T, CEM, H9 and MT-4) using the Absolutely RNA MiniPrep Kit from Stratagene, following the manufacturer’s instructions. RNA concentration and purity was determined using the Nanodrop spectrophotometer (Thermo Fisher Scientific).

### RT-qPCR

The expression of syndecan 1–3, glypican 1–6, serglycin and the reference genes glyceraldehyde 3-phosphate dehydrogenase (GAPDH) and β-actin in the different lymphocytes and cell lines was analyzed using QuantiTect Primer Assays (QIAGEN, Oslo, Norway) (Table 1). The primer pair used for syndecan-4 (forward 5′-ACGATGAGGATGTAGTGGGG – 3′, reverse 5′TAGTTTCTTGGGTTCGGTGG - 3′) was synthesized from Operon Biotechnologies, Germany. cDNA was synthesized from total RNA (30 ng from each lymphocyte sample and 500 ng from each cell line sample) using the QuantiTect Reverse Transcription Kit (QIAGEN, Oslo, Norway). Reverse transcription was repeated twice for each sample. PCR reactions were carried out in duplicates using SYBR Green I and the real-time PCR system Mx 3000P (Stratagene, La Jolla, CA). One ng from each cDNA sample prepared from the lymphocytes and 50 ng from each cDNA sample prepared from the cell lines were amplified with the different primers in 50 μl total reaction volume containing 25 μl 2x QuantiTect SYBR Green PCR Master Mix and 0.3 μM primers. Amplification conditions were 15 min at 95.0 °C, followed by 40 cycles of 15 s at 94.0 °C, 30 s at 55.0 °C and 30 s at 72.0 °C. At the end of each PCR run, the dissociation curves were analyzed for detection of non-specific PCR product. No template control wells were also included in each PCR run to rule out primer–dimer formation.

**Table 1:** QuantiTect primer assays

Gene	Assay	Cat.
Syndecan-1	Hs_SDC1_1_SG	QT00037128
Syndecan-2	Hs_SDC2_1_SG	QT00001001
Syndecan-3	Hs_SDC3_1_SG	QT00036386
Glypican-1	Hs_GPC1_1-SG	QT00038759
Glypican-2	Hs_GPC2_1-SG	QT00030478
Glypican-3	Hs_GPC3_1-SG	QT00051807
Glypican-4	Hs_GPC4_1-SG	QT00011109
Glypican-5	Hs_GPC5_1-SG	QT00102767
Glypican-6	Hs_GPC6_1-SG	QT00054628
Serglycin	Hs_SRGN_1_SG	QT00001225
GAPDH	Hs_GAPDH_1_SG	QT00079247
Β-actin	Hs_ACTB_1_SG	QT00095431

Differences in the quantification cycle (Cq) for the target gene and the mean Cq for the reference genes, called ΔCq, were calculated to normalize for the differences in the amount of total nucleic acid added to each reaction and for the efficiency of the reverse transcriptase step. Cq values >35 were considered negative. The ΔCq values were converted to the linear form using the term 2^-ΔCq^ in order to perform statistical analysis [25]. Expression of each target gene in the primary normal lymphocytes was analyzed in three independent peripheral blood samples. The mean 2^-ΔCq^ values were calculated from two PCR replicates per cDNA sample. The data are presented as C x 2^-ΔCq^, with C = 10_._^5^

## Results

### Gene expression of proteoglycan core proteins in normal human lymphocytes

**Fig. 1:**
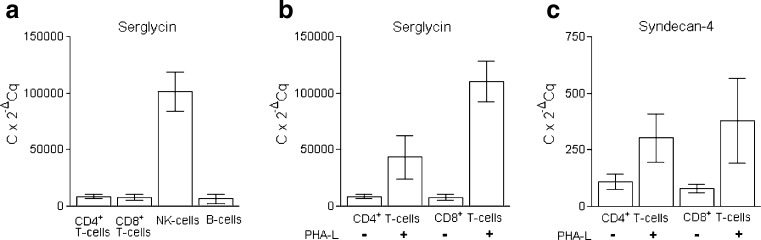
Gene expression of serglycin and syndecan-4 in lymphocytes. **a** Level of serglycin mRNA in NK cells, B-cells and CD4+ and CD8+ T-cells. **b** Level of serglycin mRNA in CD4+ and CD8+ T-cells after stimulation with 1 μg/ml PHA-L for 20 h. **c** Level of syndecan-4 mRNA in CD4+ and CD8+ T cells, and after stimulation with 1 μg/ml PHA-L for 20 h. The mean 2^-ΔCq^ values were calculated from two PCR replicates per cDNA sample. ΔCq = (Cq _target gene_– Cq _reference gene_). The data are presented as C x 2^-ΔCq^, with C = 10^5^

CD4+ and CD8+ T-cells, NK-cells and B-cells were isolated from peripheral blood by cell sorting and total RNA was analyzed by quantitative PCR in order to determine the expression levels of syndecans, glypicans and serglycin. As shown in Fig. 1a, all four cell types contained mRNA encoding serglycin. B cells, CD4+ and CD8+ cells contained approximately equal amounts of mRNA, whereas NK cells contained approximately 13 fold more serglycin mRNA compared to the B-cells, CD4+ and CD8+ cells.

To investigate the effects of mitotic stimulation on serglycin gene expression, CD4+ and CD8+ T-cells were incubated with Phytohemagglutinin-L (PHA-L), which is known to bind the T-cell receptor complex and trigger expression of genes that control cell growth and immune effector functions [26]. Stimulation of T-cells with PHA-L for 20 h resulted in a 5 and 15 fold increase in serglycin mRNA expression in CD4+ and CD8+ T-cells, respectively (Fig. 1b).

The NK-cells and B-cells did not express mRNA encoding syndecans or glypicans. However, both CD4+ and CD8+ T-cells contained mRNA for syndecan-4 in approximately equal amounts. Furthermore, after PHA-L stimulation the CD4+ and CD8+ T-cells increased their expression of mRNA encoding syndecan-4 about 3 and 8 fold, respectively (Fig. 1c), but had no effect on the expression of other syndecans or glypicans. The results from the RT-qPCR indicate that the mRNA level for serglycin is higher than for syndecan-4 in both CD4+ and CD8+ T-cells (Fig. 1).

### Biosynthesis of proteoglycans in normal lymphocytes

**Fig. 2:**
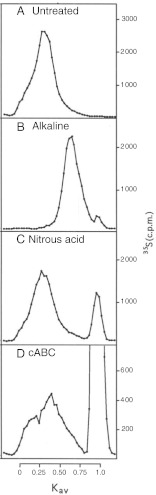
Superose 6 gel chromatography. Lymphocytes cultures were labelled with [^35^ S]sulfate for 24 h. Radiolabeled macromolecules were isolated from the culture medium and analysed by Superose 6 gel chromatography before **a** and after treatment with alkaline borohydride **b**, nitrous acid **c**, or chondroitinase ABC **d**

**Fig. 3:**
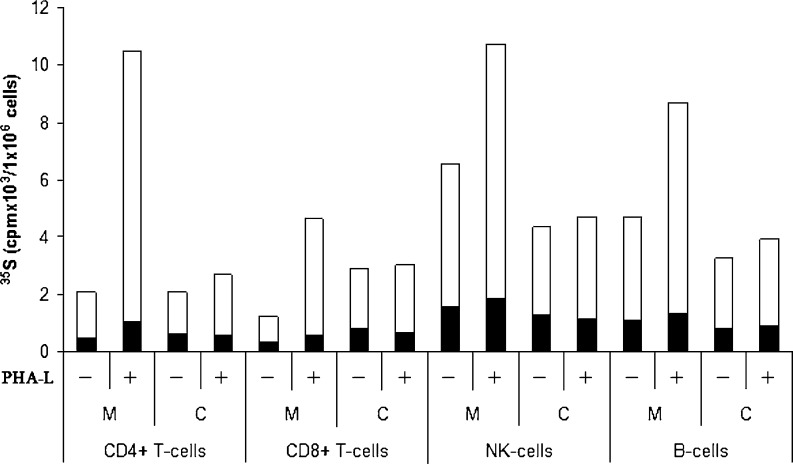
Biosynthesis of [^35^ S]CS and HS in different subtypes of normal lymphocytes. Purified lymphocytes were cultured *in vitro* with and without PHA-L and labeled with ^35^ S-sulfate. ^35^ S-labeled macromolecules from medium (M) and cell (C) fractions were analyzed by gel chromatography after Chondroitinase-ABC and heparitinase or HNO2 treatment to determine the amount of CS (*white*) and HS (*black*). The incorporation into ^35^ S-labeled macromolecules is expressed per one million cells for all cell types used. The experiment was repeated three times and the results presented are from one representative experiment

To analyze the PG biosynthesis in NK cells, B-cells and CD4+ and CD8+ T-cells, all cell types were cultured and labelled with [^35^ S]sulfate. Incubation of cells with [^35^ S]sulfate will lead to incorporation of [^35^ S]sulfate into newly synthesized GAGs attached to proteins in the golgi complex [27]. The ^35^ S-labeled macromolecules from medium and cell fractions were harvested and analyzed by Superose 6 gel chromatography prior to and after alkaline borohydride treatment, which liberate the GAG chains from the core protein. These investigations showed that ^35^ S-labeled macromolecules in the culture medium from all four cell types were part of PG molecules (Fig. 2), whereas the cell fractions contained a mixture of intact PGs and partly depolymerized GAGs (results not shown). Aliquots of the respective fractions containing ^35^ S-labeled macromolecules were also digested with chondroitinase ABC (depolymerizes CS and dermatan sulfate (DS)), heparitinase (depolymerizes HS) or nitrous acid (cleaves N-sulfated regions found in heparin/HS), and subsequently analyzed by gel chromatography to quantify the amount of CS and HS. More than 95 % of the ^35^ S-labeled macromolecules isolated both from the culture medium and the cell fraction were depolymerized by a combination of chondroitinase ABC and heparitinase, demonstrating that virtually all the ^35^ S-labeled macromolecules were PGs/GAGs (results not shown). Data from gel chromatography were used to calculate the amount of synthesized [^35^ S]CS and [^35^ S]HS (Fig. 3). All four subtypes of lymphocytes synthesized a mixture of HS and CS. In unstimulated cells, 70–80 % of the ^35^ S-labeled macromolecules isolated from both the culture medium and the cell fractions were CS and the remainder HS. Stimulation of the cells with PHA-L increased the expression of [^35^ S]PGs. This was mostly due to increased secretion of CS PG into the culture medium, varying from 2 to 6 fold increase in the different cell types compared with control. Only minor increases were observed for the HS counterpart. The relative increase in PG secretion after stimulation was highest in CD4+ T-cells, followed by CD8+ T-cells, B-cells and NK-cells. The content of CS and HS in the cell fractions was either unaffected, or just slightly increased, by PHA-L treatment. NK-cells and B-cells showed a higher ^35^ S-incorporation (2–3 fold) than CD4+ and CD8+ T-cells, reflecting a higher level of PG biosynthesis in these cells. Furthermore, CD8+ T-cells secreted a lower proportion of newly synthesized PGs compared to CD4+ T-cells, NK-cells and B-cells. Approximately 70 % of the radiolabeled PGs in unstimulated CD8+ T-cells were found in the cell fraction after 20 h incubation, compared with 40–50 % in CD4+ T-cells, NK-cells and B-cells, indicating that more PGs are associated with storage granules in the CD8+ T-cells [28] . From Fig. 3 it can also be noted that NK-cells had the highest level of cell-associated PGs of all the primary cells tested. These cells also had the highest level of serglycin mRNA as shown in Fig. 1a.

### Cell surface proteoglycans on normal lymphoid cells

**Fig. 4:**
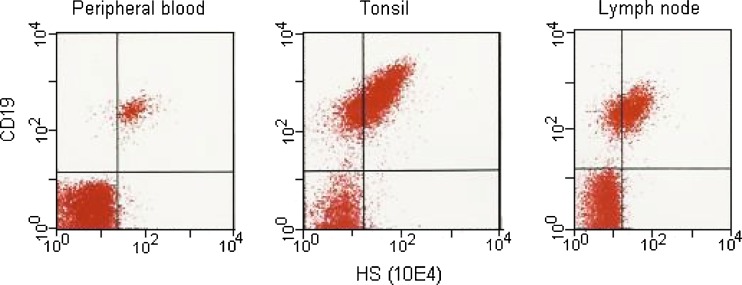
Flow cytometry of B-cells from different tissues B-cells from peripheral blood, tonsils and lymph nodes were subjected to flow cytometry using antibodies against CD19 and HS (10E4)

To investigate the presence of cell surface HS, flow cytometry was performed on various human lymphoid cells using antibodies against HS (10E4). The 10E4 antibodies react with an epitope that occurs in native HS chains and that is destroyed by N-desulfation of the glycosaminoglycan [29]. NK cells, CD4+ and CD8+ T-cells were all negative for the presence of cell surface HS. However, B-cells (expressing CD19 as marker) were shown to be positive for the presence of HS, both in cells isolated from peripheral blood, tonsils and lymph nodes (Fig. 4). No syndecan-1 was detected on any of these cell types.

### Gene expression of proteoglycan core proteins in lymphoma and leukemia cell lines

To further investigate the expression of PGs in human lymphoid cells, RT-qPCR analyses were performed on total RNA isolated from human lymphoma and leukemia cell lines. All cell lines contained mRNA encoding serglycin. However, the two T-cell lines H9 and MT-4 expressed the highest level of mRNA encoding serglycin, while the two B-cell lines Ramos and KMS-5 displayed a low level of the corresponding mRNA (Table 2).

**Table 2:** Levels of mRNA encoding cell surface proteoglycans and serglycin in different lymphoma/leukemia cell lines

B cell lines						T cell lines			
	Namalwa	Ramos	Sudhl-6	U-266	KMS-5	Sup-T	CEM	H9	MT-4
SDC1	0.9			2093.2	1.6		583.4	0.2	0.9
SDC2				4.1	658.2				
SDC3					44.3				
SDC4			112.7		375.3	0.4	215.3	291.6	403.7
GPC1			4.7		17.5	4.3	123.2	0.6	28.2
GPC2	19.4	34.3	15.3		32.1	45.1	70.6	45.1	11.8
GPC3						58.4			
GPC4									
GPC5	8.3								
GPC6					256.2		8.6		4.2
SRGN	2246.1	13.5	3673.9	9704.9	39.3	504.5	658.6	13205	15387

The mean 2^-ΔCq^ values were calculated from two PCR replicates for each cDNA sample

ΔCq = (Cq _target gene_– Cq _reference gene_)

The data are presented as C x 2^-ΔCq^, with C = 10^5^

All cell lines expressed mRNA encoding one or several types of syndecans, except the B-cell line Ramos (Table 2). Furthermore, all cell lines expressed mRNA for one or several types of glypicans. Three of the B-cell lines and three of the T-cell lines expressed mRNA encoding syndecan-1; but with great variation in the amount of syndecan mRNA expressed. Expression of mRNA encoding syndecan-2 and -3 was only found in the myeloma cell lines U-266 and KMS-5, however the U-266 cells showed a low expression of syndecan-2 mRNA compared to KMS-5 and no expression of syndecan-3 mRNA. All the T-cell lines, in addition to two B-cell lines expressed mRNA encoding syndecan-4 and glypican-1. Furthermore the expression of mRNA encoding glypican-2 was found in all the cell lines, except for the myeloma cell line U-266. Glypican-6 mRNA was found in one B-cell line and two T-cells lines, where the KMS-5 cells displayed the highest expression. Glypican-3 and -5 mRNA was only found in one T-cell line and one B-cell line, respectively. Taken together, those cell surface PGs mostly expressed were glypican-2 (in 8 of 9 cell lines), glypican-1, syndecan-1 and syndecan-4 (in 6 of 9 cell lines for all three). Serglycin, in contrast, was expressed in all the cell lines.

### Biosynthesis of proteoglycans in lymphoma and leukemia cell lines

**Fig. 5:**
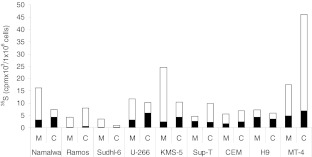
Biosynthesis of [^35^ S]CS and HS in different lymphoma and leukemia cell lines B- and T- cell lines were cultured and labeled with [^35^ S]sulfate for 20 h. ^35^ S-labeled macromolecules from medium (M) and cell (C) fractions were analyzed by gel chromatography after Chondroitinase-ABC and HNO2 treatment to determine the amount of CS (*white*) and HS (*black*) in each fraction. The incorporation into ^35^ S-macromolecules is expressed per one million cells for all cell lines used. The experiment was repeated three times and the results presented are from one representative experiment

**Fig. 6:**
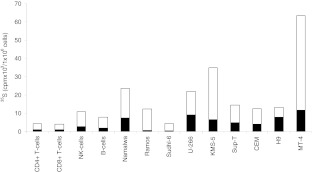
Biosynthesis of [^35^ S]CS and HS in different subtypes of normal lymphocytes and in lymphoma and leukemia cell lines. Total amount (medium and cell fraction summarized) of [^35^ S]CS (*white*) and [^35^ S]HS (*black*) synthesized by the different cells

The expression of PGs in the cell lines was also studied by labeling with [^35^ S]sulfate for 20 h and harvesting conditioned media and cell fractions as described above. As for the primary lymphocytes, all the cell lines synthesized both HS and CS PGs, which were partly secreted into the culture medium (Fig. 5). Furthermore, in these cells the major part of the GAGs was of the CS type. One exception was the T-cell line H9 which synthesized more HS than compared to CS. By comparing the synthesis of HS and CS in the cell lines with the normal cells it was clear that nearly all the cell lines synthesized more HS and CS than the primary, normal lymphoid cells (Fig. 6).

### Cell surface proteoglycans on lymphoma and leukemia cell lines

Flow cytometry was also used to investigate if these human cell lines contained cell surface HS or syndecan-1. As shown in Table 3, three of the B-cell lines, Namalwa, U-266 and KMS-5 expressed both syndecan-1 and HS on the cell surface. Also two T-cell lines, MT4 and H9 stained positive for syndecan-1 and HS expression on the cell surface. The CEM cell line, which displayed the highest level of syndecan-1 mRNA among the T-cells lines, was negative for syndecan-1 expression and HS expression on the cell surface, indicating a post-transcriptional down-regulation.

**Table 3:** Presence of cell surface HS proteoglycans on B and T cell lines

	Cell type	^a^Syndecan-1	^b^HS
B-cells lines	Namalwa	+	+
	Ramos	−	−
	Sudhl-6	−	−
	U-266	+	+
	KMS-5	+	+
T cell lines	Sup-T	−	−
	CEM	−	−
	H9	−/+	−/+
	MT-4	+	+

^a^Surface expression was detected by flow cytometry using antibody against syndecan-1 (CD138)

^b^Surface expression was detected by flow cytometry using antibody against HS (10E4)

+: strongly positive;−/+: positive, but low intensity (dim);−: negative

### Serglycin expression in lymphoma and leukemia cell lines

**Fig. 7:**
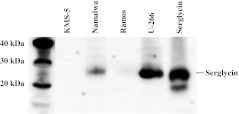
Expression of serglycin in lymphoma and leukemia cell lines. Immunoblot of cell medium from KMS-5, Namalwa, Ramos and U-266 cell lines probed with anti-serglycin

To determine if the expression of serglycin mRNA correlated with the expression of serglycin core protein, serum free media from KMS-5, U-266, Namalwa and Ramos were analysed by Western blotting (Fig. 7). These four cell lines expressed highly different amount of serglycin mRNA, as shown in Table 2. The Western blot analysis revealed that the culture medium from U-266 cells contained more serglycin than the culture medium from Namalwa, which correlate with the mRNA levels. The culture medium from KMS-5 and Ramos did not contain detectable amounts of serglycin. These two cell lines also expressed much less serglycin mRNA than the other cell lines, as shown in Table 2. This shows that the expression level of serglycin mRNA corresponds very well to the expression level of serglycin core protein.

## Discussion

In the present study we have investigated the expression of serglycin and cell surface PGs in B-cells, CD4+ T-cells, CD8+ T-cells and NK cells. We have also compared the expression of these PGs in different lymphoma and leukemia cell lines. The results presented show that mRNA encoding serglycin core protein was detected in all cells, while the expression of core proteins for cell surface PGs varied to a great extent in the different cell types. The expression of serglycin in normal B-cells, as well as the high expression of serglycin in NK cells, compared to T-cells, is novel information and should merit further studies on the functions of serglycin in these sub-populations of lymphocytes. Studies on serglycin functions in cytotoxic T-cells have shown that serglycin is used as a storage scaffold for granzyme B [30,31], a serine protease also present in cytotoxic granules in NK cells [32]. It has also been reported that resting mouse NK cells have abundance of granzyme B transcripts, but not of the protein. However, once the cells were activated there was a significant increase in granzyme B protein levels [33]. In contrast, T-cells upregulate the transcription of granzyme B after activation [34]. By treating the T-cells with PHA-L we found that the amount of serglycin mRNA increased in the T-cells, especially in the CD8+ T-cells, showing that activation also induces the transcription of serglycin. One hypothesis for the higher expression of serglycin mRNA in the NK cells compared to the T-cells could therefore be that the expression of serglycin is regulated more at the transcriptional level in T-cells, compared to post-transcriptional level in the NK-cells.

We could not detect mRNA encoding syndecans or glypicans in normal NK-cells and B-cells. However, both CD4+ and CD8+ T-cells isolated from peripheral blood expressed syndecan-4 mRNA. Recently, syndecan-4 was shown to be involved in inhibition of T-cell activation, where syndecan-4 expression was increased upon activation of the T-cells [14,15,35], supporting our finding that activation of T-cells by PHA-L increased the level of mRNA encoding syndecan-4. CD4+ T-cells have been reported to express low levels of mRNA encoding syndecan-2 [15]. Stimulating the CD4+ and CD8+ T-cells with PHA-L did not induce mRNA expression of other members of the syndecan or the glypican families. This is in accordance with previous findings, showing that PHA-L stimulation of T-cells did not induce expression of syndecan-1, 2, or 3 [35]. In contrast, PMA/ionomycin stimulation of T-cells induced syndecan-1 mRNA expression [35], showing that gene expression of the different syndecans depends on the type of T-cell activator used probably reflecting the various signal transduction pathway activated by different stimuli.

We found that the major GAG synthesized by normal lymphoid cells was CS. This support that serglycin is more abundant in these cells compared with syndecans and glypicans, since these PGs mainly are substituted with HS. The NK-cells produced the highest amount of CS PG, which correlates with the much higher amount of serglycin mRNA in these cells compared to the other lymphocyte subtypes. However, the PHA-L induced increase in the synthesis and secretion of CS PG, presumably serglycin, was much higher in CD4+ and CD8+ T-cells than in NK- and B-cells. PHA-L is a mitogen which is known to stimulate T-cells, whereas NK-cells and B-cells are not reported to be significantly activated by PHA-L [36,37]. It was therefore not expected to be a large increase in expression of proteoglycan in these cells after PHA-L exposure. Most of the synthesized PGs/GAGs in NK-cells were not secreted, but associated with the cells. NK-cells have a large number of secretory granules and it is known that secretory granules in mast cells contain high amounts of serglycin [38]. The cell-associated PGs/GAGs found in the NK-cells may therefore very well be serglycin located in secretory granules. In CD8+ T-cells there was a discrepancy between the fold change of serglycin mRNA and fold change of secreted PGs after PHA-L stimulation (Figs. 1 and 3). This discrepancy may be due to change in degree of sulfation or length/number of GAG chains after stimulation. It could also be due to post-transcriptional regulation or increased intracellular turnover.

Despite the lack of mRNA encoding syndecans or glypicans, both NK-cells and B-cells synthesized HS. The HS chains must therefore be attached to other PG core proteins in these cells. In mast cells, serglycin can be substituted with heparin/HS. It is therefore likely that at least some of the HS is linked to serglycin in NK- and B-cells. In mice, it has been shown that syndecan-1 is expressed on pre-B-lymphocytes within the bone marrow, is absent on circulating and peripheral B-cells, and is re-expressed on mature plasma cells [17]. In our experiments we found that HS was present on the surface of normal B-cells in peripheral blood and in a fraction of the B-cells in tonsils and lymph nodes. Since we could not detect mRNA expressing syndecans or glypicans in these cells, the HS molecules must be linked to other PG core proteins on the cell surface of these cells or associated with the cell surface through other types of interactions. In contrast to the NK- and B-cells, the CD4+ and CD8+ T-cells expressed mRNA encoding syndecan-4. Some of the HS in these cells may therefore be attached to syndecan-4.

It has been suggested that, in addition to CD4 molecules and chemokine receptors, cell surface HS PGs participate in the binding of human immunodeficiency virus (HIV) to T-lymphocytes [39–41]. These reports were based on studies of cell lines expressing HS at the cell surface, questioning the biological relevance. In the present study we could not detect any HS on the cell surface of the CD4+ T-cells by flow cytometry, using the anti-HS antibody 10E4. However, by labeling the cells with [^35^ S]sulfate we found that normal CD4+ T-cells, isolated from peripheral blood, did express cell-associated HS PG. Further, the cells expressed mRNA encoding syndecan-4 which was 3-fold upregulated in activated CD4+ T-cells. This may indicate that persons having activated T-cells are more susceptible for HIV-infection.

We found a large variation in the expression of PGs in the different lymphoma and leukemia cell lines. In contrast to serglycin which was expressed in all the cells, the presence of mRNA encoding syndecans and glypicans varied between the different cell lines. Interestingly, there was also a large difference between the two cell lines, U-266 and KMS-5, both obtained from patients with multiple myeloma. This shows that the expression of a distinct PG may differ among cell lines established from patients with the same type of lymphoma. The higher synthesis of CS and HS molecules in the T-cell lines compared to the normal T-cells suggests that malignant transformation of T-cells induces increased synthesis of PGs, especially of the cell surface types. Studies on surgical biopsies from patients are needed to determine if expression of various PGs can be used as a diagnostic tool to discriminate between different entities of non-Hodgkin lymphomas. In summary, our results show that serglycin was the dominant PG expressed by normal B- and T-lymphocytes. Serglycin was also a major PG in the malignant lymphoid cells, but these cells also expressed one or more types of cell surface PGs.
